# My Grief App for Prolonged Grief in Bereaved Parents: A Pilot Study

**DOI:** 10.3389/fpsyt.2022.872314

**Published:** 2022-04-25

**Authors:** Rakel Eklund, Maarten C. Eisma, Paul A. Boelen, Filip K. Arnberg, Josefin Sveen

**Affiliations:** ^1^Department of Medical Sciences, National Centre for Disaster Psychiatry, Uppsala University, Uppsala, Sweden; ^2^Department of Clinical Psychology and Experimental Psychopathology, University of Groningen, Groningen, Netherlands; ^3^Department of Clinical Psychology, Utrecht University, Utrecht, Netherlands; ^4^ARQ National Psychotrauma Centre, Diemen, Netherlands

**Keywords:** bereavement, intervention, parents, pediatrics, prolonged grief, smartphone application

## Abstract

The death of a child is a devastating experience for most parents. Consequently, bereaved parents are at risk to develop physical and mental health problems, including prolonged grief disorder. Nevertheless, there is a lack of evaluated psychosocial interventions for bereaved parents. The primary aim of this study was to examine the feasibility of the My Grief app for bereaved parents. The secondary aim was to evaluate the potential reduction of symptoms of prolonged grief, depression and post-traumatic stress, and cognitive-behavioral processes proposed to prolong grief reactions. The study was a mixed-method open trial design, using pre- and post-intervention surveys and post-intervention interviews. Thirteen parents had access to the app for 4 weeks, eight parents participated in interviews and 10 parents answered the follow-up survey. The study provided evidence for the app's feasibility and acceptability, with participants reporting satisfaction with the app and stating that they would recommend it to parents in similar situations. According to the participants, the app was easy to use, the content gave a feeling of not being alone or weird in how one grieves, and the app gave a valuable overview of information, knowledge and further support. In addition, all parents expressed that an app like My Grief is needed and would be particularly useful to access early in the grieving process. Significant reductions of prolonged grief symptoms (*d*_*within*_ = 0.86) and grief-related rumination (*d*_*within*_ = 0.72), loss avoidance (*d*_*within*_ = 0.95) and negative cognitions (*d*_*within*_ = 1.36) from pre- to post-assessment were found. In conclusion, the app appears acceptable and feasible to use and will be evaluated in a larger randomized controlled trial (Trial registration number: NCT04552717, https://clinicaltrials.gov/ct2/show/NCT04552717).

## Introduction

The death of a child is one of the most devastating events that a parent can experience. Most bereaved people come to terms with the loss without requiring professional help or support. Common experiences in the initial month following the loss of a loved one are sadness, yearning, anxiety, and feelings of emptiness. Generally, these feelings decrease over time as the bereaved person adjusts to the loss (1). However, several studies show that a substantial subset of bereaved parents develops various physical and psychological health problems that persist many years after the loss (2, 3). Bereaved parents are also at a heightened risk for developing persistent, severe and disabling grief, commonly termed prolonged grief or complicated grief (4, 5). Recently, diagnostic handbooks have recognized prolonged grief disorder (PGD) as a formal diagnosis. It is characterized by persistent and pervasive yearning for the deceased, persistent and pervasive cognitive preoccupation with the deceased, as well as symptoms of intense emotional pain, such as difficulty accepting the loss, a feeling that one has lost a part of one's self, and difficulty in engaging in social activities (6). One study found that 16% of parents bereaved of a child due to cancer showed responses signaling probable PGD (7).

Despite the potentially severe consequences of losing a child, there is a lack of evaluated psychosocial interventions for bereaved parents (8, 9). However, cognitive-behavioral therapy (CBT) is generally shown to be effective in treating prolonged grief symptoms both face-to-face (10–13) or internet-based formats in bereaved adults (14–16). CBT treatment for PGD includes creating a coherent, meaningful narrative of oneself regarding the loss, challenging negative beliefs about the self, the world and the future, and gradually confronting avoided reminders of the loss such as places, memories, or objects. It also includes setting new life goals and stimulating engagement in meaningful activities (17). Some studies have focused on using complementary therapeutic techniques (e.g., mindfulness and relaxation exercises) to ameliorate prolonged grief symptoms (18–20).

Self-help applications in smartphones (apps) have several advantages over face-to-face and web-based intervention, such as their availability, accessibility, the immediate support they can provide, as well as their anonymity, low costs, and the possibility of tailoring it to the user (21). Thus, mobile apps have a great potential to be used as self-help interventions for stress-related mental health problems. A literature review of the effectiveness of mobile apps for mental health problems has shown that using mobile apps can decrease different mental health problems, such as depression, anxiety, substance abuse, sleeping disorder and posttraumatic stress disorder (PTSD) (22). For example, one app focused on reducing mental health problems following major negative life events is the *PTSD Coach*, a self-management app focused on improving knowledge of PTSD symptoms and providing support for trauma-related distress (23). A recent randomized controlled trial (RCT) on the Swedish PTSD Coach showed a reduction in posttraumatic stress and depression symptoms compared to a waitlist control group (24). Several studies on the PTSD Coach further show that the participants generally experience no or few adverse negative effects of using the app and support the acceptability and feasibility of using the app (24–27).

Based on existing knowledge, we believe that an app targeting bereaved parents who lost a child, using elements of CBT, could potentially be effective in improving mental health in those parents. To our knowledge, no apps for bereaved individuals have yet been empirically examined. This pilot study aimed to elucidate the feasibility of the app My Grief in bereaved parents. A secondary aim was to provide a preliminary evaluation of the potential effect of using the app for 1 month on prolonged grief, depressive and posttraumatic stress symptoms, and cognitive-behavioral processes proposed to perpetuate grief reactions (i.e., grief rumination, anxious and depressive avoidance, and negative grief cognitions).

## Materials and Methods

### Design

This study is part of a larger study aimed to evaluate My Grief for parents who lost a child (28). The present study evaluates the parent's use of the app for 4 weeks. Four weeks of app access was considered sufficient to evaluate the feasibility of the intervention based on a previous pilot-study of PTSD Coach (25). The study has an open trial mixed-method design, using pre- and post-intervention surveys and post-intervention interviews.

### Intervention—The Self-Management App My Grief

**Figure 1:**
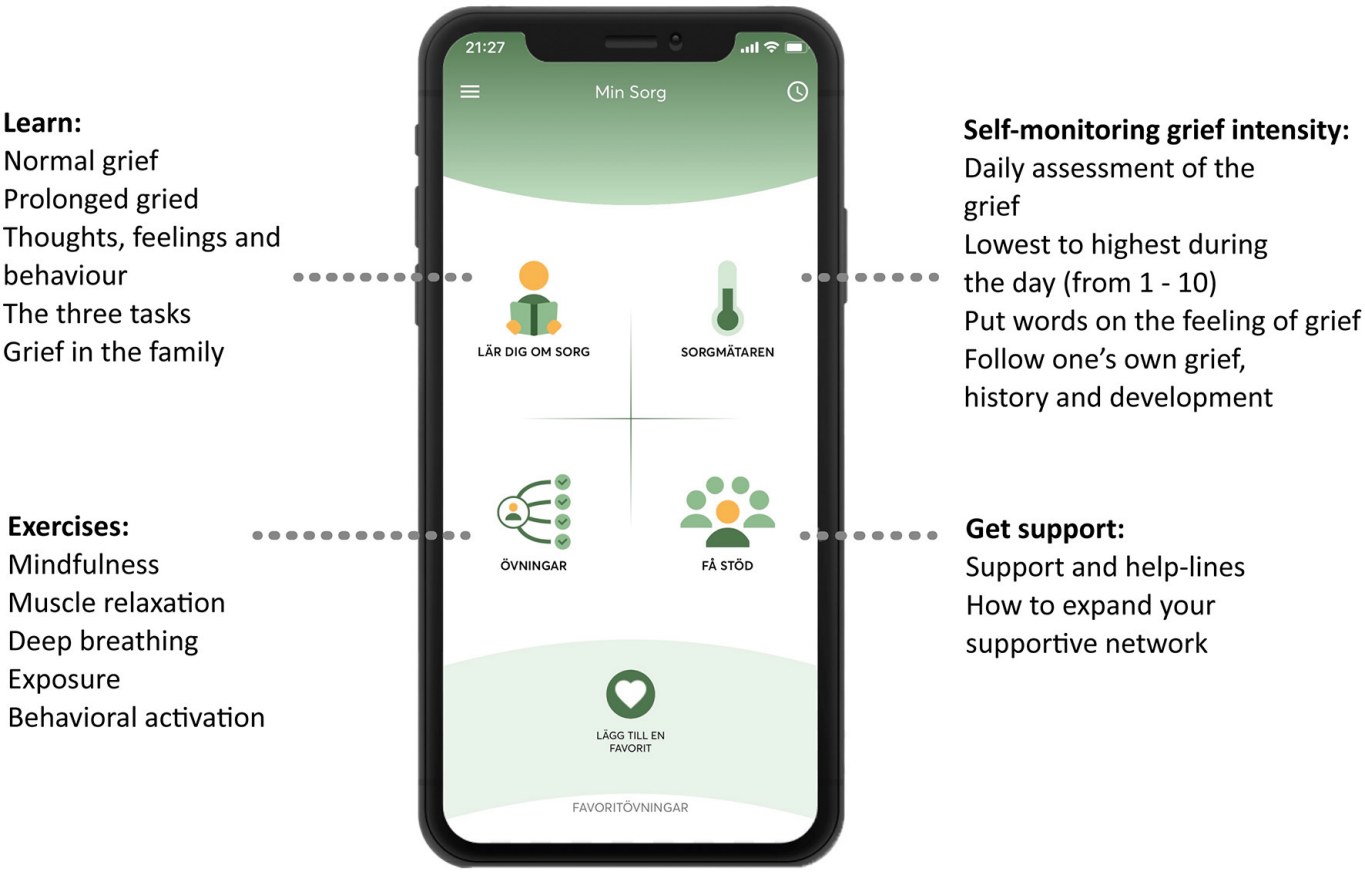
Structure of the *My Grief* app.

This intervention entailed receiving access to a self-management app called My Grief (in Swedish called *Min Sorg*), available for smartphone users (iOS and Android), in Swedish. The content in the app is based on CBT principles, and a cognitive-behavioral conceptualization of prolonged grief, which includes three tasks: (1) Facing the loss and the pain that goes with it, (2) keeping confidence in yourself, others, life and future, and (3) engaging in activities that may promote adjustment to the loss (10, 29). The app is also based on an online PGD-treatment manual (30, 31). The structure of the app is based on the PTSD Coach and parts of the self-guided exercises are from the Swedish version of the PTSD Coach. My Grief includes four sections (see Figure 1), which are: (1) *Learn*—psychoeducation about grief, (2) *Self-monitoring of grief intensity*—a rating scale to assess the daily intensity of the grief, (3) *Exercises*—self-guided exercises, both recorded audio exercises (e.g., mindfulness exercises) as well as writing exercises (e.g., exposure to memories of the loss) and (4) *Get support*—contact details to different support functions and help-lines, and psychoeducation regarding expanding one's social network. For further information about the content, structure, and exercises of the app, see the study protocol (28).

### Settings and Participants

Parents were recruited from the Swedish Childhood Cancer foundation via their social media sites during April 2021. Inclusion criteria for participation were being a parent of a child who died at least 1 year ago, having mild to severe symptom levels of prolonged grief [a cut-off of >16 on the PG-13 was chosen to include parents with elevated PGD symptoms, as individuals with mild to moderate symptoms could benefit from the intervention (24)], understanding and speaking Swedish, and having access to a smartphone. Exclusion criteria were self-reported current suicidal thoughts or psychosis, assessed with single items in the screening questionnaire. The parents signed up for the study via a website, www.minsorg.com, which automatically directed the parents to a screening questionnaire and, if they were eligible, to the informed consent form and the pre-intervention survey, all at an online platform hosted by Uppsala University.

**Figure 2:**
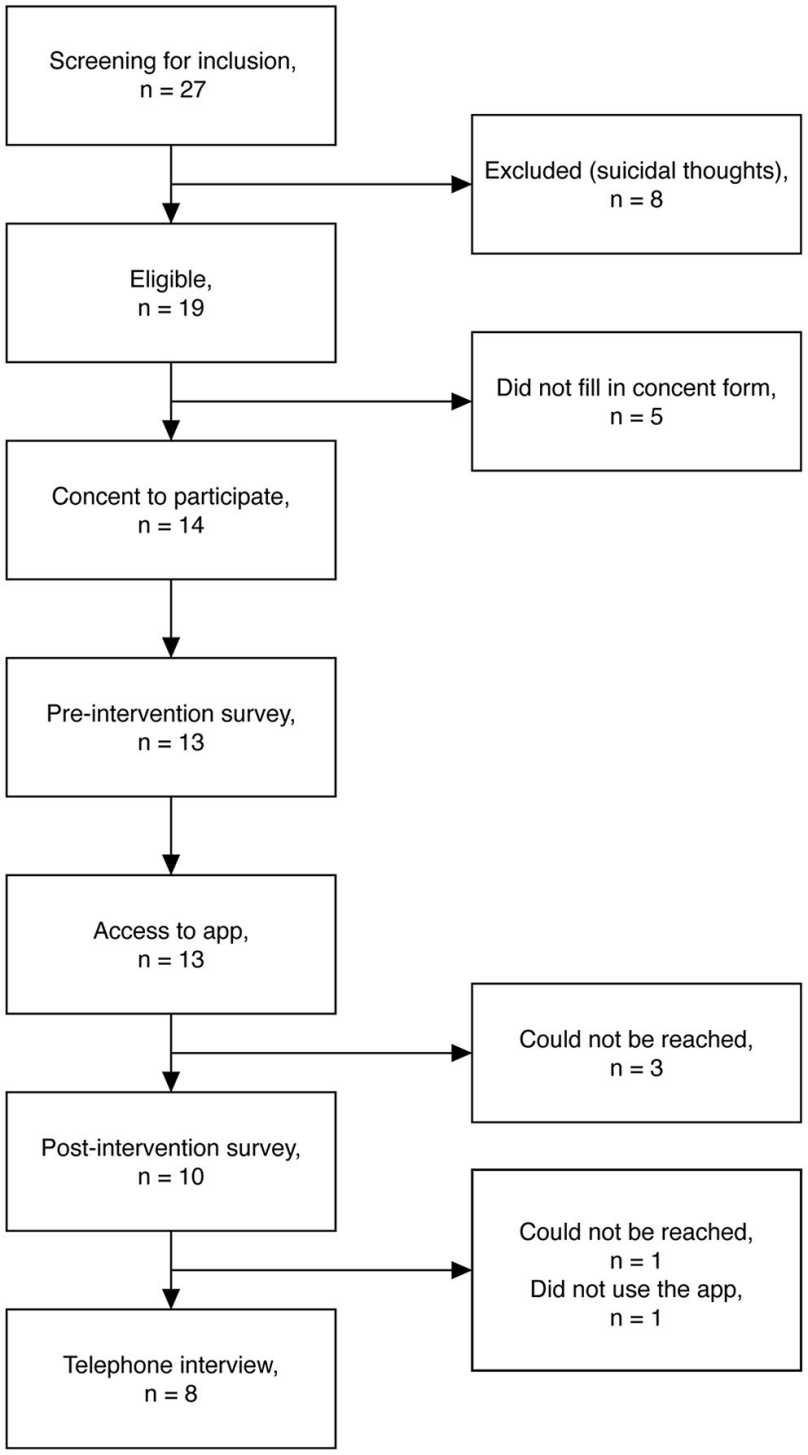
Flowchart over recruitment and pre-post assessments.

A total of 27 parents filled in the screening survey, to assess if they were eligible to participate in the study, whereof eight parents were excluded due to having suicidal thoughts [for full inclusion and exclusion criteria: (28)]. Of the 19 eligible participants, five did not give consent to participate. Of the 14 who consented to participate, 13 parents completed the pre-intervention survey and thereafter got access to the My Grief app for 4 weeks. One week after enrolment, a researcher from the research group called the parents by phone, to check that everything was working out properly with the app, to answer any questions about the app or the study, and to set up a time for a telephone interview. After 4 weeks, 10 parents filled in the post-intervention survey and eight parents were interviewed by telephone; these 10 and eight parents were included in the quantitative and qualitative analyses, respectively (Figure 2 shows a flowchart).

### Ethical Approval

The study has received ethical approval from the Swedish Ethical Review Authority (project no. 2020-01704).

### Data Collection

#### Interviews

Semi-structured telephone interviews were conducted by one of the researchers (RE), 4 weeks after the parents had received access to My Grief. The aim of the interviews was to elucidate how the parents had used the app, which parts in the app were used the most and least, and to receive suggestions for improvements (see interview guide, Supplementary Material). Eight parents participated in the interviews. Five remaining parents did not want to participate as they indicated they did not use the app that much (*n* = 1) or they could not be reached (*n* = 4). The interviews lasted between 14 and 45 min, were audio-recorded, and transcribed verbatim.

#### Survey

The survey consisted of questionnaires assessing prolonged grief, depression and posttraumatic stress symptoms, cognitive-behavioral variables (i.e., grief rumination, anxious and depressive avoidance, negative grief cognitions) as well as feasibility and user experiences of the app.

Prolonged grief was measured by Prolonged Grief Disorder-13 (PG-13). It consists of 13 items including 11 items assessing cognitive, behavioral and emotional symptoms during the past month, rated on a 5-point scale (not at all—several times a day/overwhelmingly). It also contains two items on duration and impairment (yes/no). PG-13 is scored by adding scores from the 11 items assessing symptoms. The total score ranges from 11 to 55, with higher scores indicating more severe prolonged grief symptoms [based on PGD criteria per (32)]. The instrument has been validated in a Swedish sample with bereaved parents and was demonstrated to have satisfactory psychometric properties and a preliminary cutoff score of ≥ 35 indicating probable PGD (7).

Depression symptoms were measured with the Patient Health Questionnaire (PHQ-9), which consists of nine items describing symptoms of depression. Participants rate to what extent they experience these symptoms during the last 2 weeks on a 4-point scale (not at all—nearly every day, 0–3). The total score ranges from 0 to 27. A higher score indicates greater symptom severity and a cutoff score of ≥ 10 may indicate probable depression (33, 34).

Posttraumatic stress disorder symptoms were measured by the Posttraumatic Stress Disorder Checklist for DSM-5 (PCL-5). It consists of 20 items describing symptoms of posttraumatic stress. Participants rate on a 5-point scale (not at all—extremely, 0–4) to what extent they experience symptoms. The total score ranges from 0 to 80, and a higher score indicates more symptoms of posttraumatic stress (35) and a total sum of ≥31–33 points may indicate probable PTSD (36). The Swedish version has shown satisfactory psychometric properties (37).

Grief-specific rumination was measured by the Utrecht Grief Rumination Scale (UGRS). The instrument consists of 15 items measuring different aspects of grief rumination. The participants' rate on a 5-point scale (never-very often, 1–5) how often they experienced certain thoughts over the last month. Total scores range from 15 to 75 and generate an overall grief rumination score (38). The instrument is validated in a Swedish sample with bereaved parents and indicates satisfactory psychometric properties (39).

Grief-specific avoidance was measured with the Depressive and Anxious Avoidance in Prolonged Grief Questionnaire (DAAPGQ). The questionnaire consists of nine items; five items measure depressive avoidance of activities and four items measures anxious avoidance of cues reminding of the loss. Participants rate on an 8-point scale (not at all true for me—completely true for me, 0–7) to what extent they engage in these avoidance strategies (40). The total score ranges from 0 to 72. The DAAPGQ was translated by two of the authors (RE and JS) following the EORTC guidelines (41).

Negative grief-cognitions were measured with the short version of Grief Cognitions Questionnaire (GCQ-18). It consists of 18 items, with statements regarding negative grief cognitions. Participants rate on a 6-point scale to what extent they agree with the statement (disagree strongly—agree strongly, 0–5). The total score ranges from 0 to 90 and a higher score indicates a stronger endorsement of negative cognitions (42). The GCQ-18 was translated by two of the authors (RE and JS) following the EORTC guidelines (41).

Eleven items from the PTSD Coach app survey (26) were revised to fit the current study, 10 items assessing the perceived helpfulness of the app, rated on a 5-point scale ranging from 0 “not at all” to 4 “extremely” and one item about the overall satisfaction of the app, rated on a 4-point scale ranging from 0 “not at all satisfied” to 3 “very satisfied.” User-experience of the app were also measured, including if the participant experienced technical problems and if the app met the participant's expectations, and if they would recommend the app to parents in a similar situation. Negative effects were assessed through the question: “Did you experience any stressful or negative consequences of using the app?” Participants who answered yes were asked to provide details in a free-text response.

### Data Analysis

A thematic analysis was used to analyze the interviews (43). The transcripts were read several times by the first and last author to get a sense of the whole and find patterns of meaning. Thereafter, the first author coded the interviews, and then a collaborative reflective process took part between the first and last author with a focus on the aims of the study and the research questions. When the dataset was coded and all codes were compared and discussed between the first and last author, the first author grouped the data into preliminary themes based on similarities and differences in the data. Next, the analysis process was conducted jointly. The analysis resulted in two themes “*experiences of the different sections and their content*” and “*the overall use and experience of the app*” with ten codes for identified themes.

Descriptive statistics were used to summarize the socio-demographic variables and to summarize the quantitative findings. The Wilcoxon paired samples test (2-tailed) was used to test for differences in prolonged grief, depression, posttraumatic stress symptoms, and rumination, negative grief cognitions and anxious and depressive avoidance, between the two assessments. Within-group effect sizes, Cohen's *d*, corrected for the correlation between paired samples (44), were calculated as a metric of the standardized mean difference between the pre- and the post-assessments. SPSS Version 28 was used for the quantitative data analysis.

## Results

### Participant Characteristics

Of the 10 participants included in the analyses, nine were women and one was male. Demographic variables of the parents and their deceased children are presented in Table 1. At the pre-assessment, the average levels of prolonged grief were relatively high (Table 2). Five of 10 participants scored above the cutoff (≥35) for probable PGD. One participant scored above the cutoff for probable depression and two participants scored above the cutoff for probable PTSD.

**Table 1:** Demographic characteristics for parents completing pre-assessment (total sample) and parents completing the post-assessment (completers).

	**Total sample (*N* = 13)**	**Completers (*N* = 10)**
**Age (years)**		
Mean (SD)	47.38 (5.79)	46.80 (6.39)
**Gender**		
Female/Male	11/2	9/1
**Place of residence**		
Large city/City/Town or smaller town/Rural area	5/2/4/2	4/2/3/1
**Country of birth**		
Sweden/Other	12/1	9/1
**Highest education level**		
University/High school/Primary school	9/3/1	8/2/0
**Current employment status**		
Working/Pregnancy leave	12/1	9/1
**Marital status**		
Married or co-living/Single	12/1	9/1
**Number of children**		
*(Including those who died)* Mean (SD)	2.92 (1.03)	2.90 (1.19)
**Lost another child**		
No/Yes	13/0	10/0
**Years from time of diagnosis**		
Mean (SD)	6.30 (3.61)	6.00 (2.66)
**Years since death^a^**		
Mean (SD)	4.81 (1.96)	4.81 (1.96)
**Diagnosis**		
Leukemia/Brain Tumor/Other	5/5/3	5/3/2

a *Data from 8 children, due to that the question were asked during the interviews and not in the baseline survey*.

**Table 2:** Mean scores, Standard deviations (SD) and Effect sizes (Cohen's d) of within-group differences for the outcome measures.

	**Total sample**	**Completers**		
	**Pre-assessment (*****N*** **=** **13)**	**Pre-assessment (*****N*** **=** **10)**	**Post-assessment** **(*****N*** **=** **10)**	**Cohen's** ***d**_***within***_*
	M (SD)	M (SD)	M (SD)	
Prolonged grief (Prolonged Grief Disorder-13)	34.6 (7.6)	34.6 (7.9) Range (min-max): 24–49	30.7 (6.0) Range (min-max): 24–41	0.86
Depression (Patient Health Questionniare-9)	8.2 (4.6)	8.1 (4.7)	7.1 (4.7)	0.44
Posttraumatic stress (Posttraumatic Stress Disorder Checklist-5)	23.5 (13.8)	21.4 (13.6)	19.4 (14.6)	0.26
Grief rumination (Utrecht Grief Rumination Scale)	48.5 (14.7)	47.0 (12.5)	40.8 (14.3)	0.72
Anxious avoidance (Depressive and Anxious Avoidance in Prolonged Grief Questionnaire)	17.0 (4.8)	17.1 (4.7)	14.7 (6.5)	0.95
Depressive avoidance (Depressive and Anxious Avoidance in Prolonged Grief Questionnaire)	20.6 (7.5)	17.9 (6.1)	16.5 (8.0)	0.37
Negative grief cognitions (Grief Cognition Questionnaire 18)	31.0 (17.5)	29.3 (16.4)	22.9 (16.9)	1.36

### Results From Interviews

#### Experiences of the Different Sections and Their Content

##### Section 1—Learn

The section regarding psychoeducation was appreciated by almost all participants. The sections about complicated grief and siblings' grief were regarded as especially good according to three of the parents. Five of the eight interviewed parents expressed gratitude to have all information gathered at one place, as one mother described it: *to have it all in one place, so good, that makes it so good. There is nowhere else, I tell you, I haven't found, I had to search through myself and participated in courses and read every single book that I came across* (mother no. 11). However, two parents, who had lost their children 5 years ago, expressed that this part of the app was not so useful to them, as they already knew a lot about grief and that this information would have been more helpful earlier in the grieving process.

##### Section 2—Self-Monitoring Grief-Intensity

Regarding the self-monitoring grief-intensity section, the parents' experiences were divided, either the parent really appreciated it or they did not. The four parents who appreciated the grief-intensity self-monitoring said that it was easy to use and that it clarified a pattern over their grief. It helped clarify for the parents that they were not always in a sad or bad mood, that some days actually could be just fine: *but there* [in the self-monitoring section] *I got the insight that yes, but today it has not been so hard and it was kind of nice sometimes to get a confirmation on it too* (Mother, no. 18). Two parents expressed that they were not in “a phase of grief” right now and that the self-monitoring was hard to use because of that. Three parents also expressed that it was difficult to use this section because they then had to think about the loss, it elicited memories. It was especially hard to fill in during the evening, just before going to sleep: *I went like into grief again at bedtime and started to think about the loss and I felt it was just so hard when I would go bed and sleep* (Mother, no. 17). Three of the eight parents reported that they used the self-monitoring section every day, while five parents used it a lot at the beginning of the 4-week study period, but less toward the end. Two parents, who had lost their children 3 and 5 years ago, suggested that this element may have been more useful at the beginning of their grieving process.

##### Section 3—Exercises

Overall, the parents appreciated the section which included different types of self-guided exercises. They were short and therefore easy to integrate into everyday life. The parents appreciated that the section included so many different exercises and the range of the offered exercises: *I think it covers a wide spectrum of exercises that are… easily accessible… and are short which you can get into everyday life… so no I think the exercises themselves are good… I think this particular section is… for me the big benefit, which I think, both now and many years ago, would have used the most and… which had given… most results* (mother, no. 17). The parents reported that they used the audio exercises several times a week. Exercises were appreciated by almost everyone. The audio exercises consisted of mindfulness training (e.g., seated practice, walking practice, awareness of the senses) and relaxation (e.g., deep breathing and body scan). It helped the parents to calm down and relax. However, two parents thought that some of the readings had a poor sound quality and therefore choose to listen to similar exercises in other apps that they already had. The audio exercises were not emotionally difficult for the parents to perform, only one parent pointed out that an exercise reminded her of the loss, in a difficult way: *with “Positive visualization”* [exercise] *I tried “Beach”* [exercise] *and it just got really hard. Well because there I have so many memories from it when we have been abroad. But, on the other hand “Forest”*[exercise] *worked, it is like that, different places are so much associated with the grief, then it can be too much* (mother, no. 11).

The writing exercises were the exercises that were most difficult for the parents to perform. They consisted of exercises of exposure, writing a narrative about the loss, and to think about future. Either they were emotionally difficult to complete or the exercises required too much peace and time to perform: *I did not do the writing … it is very difficult to find courage and peace… A lot of thoughts came to mind* (mother, no. 14). Two parents said that they recognized the writing exercises from therapy and therefore did not do them.

Two parents mentioned the reading exercises, which focused on daily activities and feelings. The parents appreciated them and reflected that they would have been especially good early in the grieving process.

##### Section 4—Get Support

Regarding the section “get support,” all parents appreciated this section. The section contained a lot of good information, and it was easy to use because links to web-pages and buttons with phone numbers were integrated. However, most parents did not use the links or numbers. Three parents had long stories during the interviews on how they had searched for this kind of information, year after years, and because of this, many of the parents told that this part of the app would have been useful early in the grieving process, one mother explained: *I have read through all the pages which… paths that you have listed… and that's great, because it was also such thing I thought was very troublesome in the beginning that even, yeah, but where the hell should I turn?* (mother, no 17).

#### The Overall Use and Experience of the App

Five of the parents used the app more frequently during the first 2 weeks of the study, and then used it less during the last 2 weeks before the interview. Three parents told that they, at first, read through all the parts of the app, then they also tested the self-monitoring section. The self-monitoring and the exercises were used daily by three parents and four of them used them just around one time a week. Two parents expressed that they did not use the app as much as they intended to.

Only one parent had a technical issue with the app when it crashed once. For everyone else, the app worked bug-free and without any errors.

The parents experienced the app as easy to use and clear, giving a calm impression. Three parents told that the green color in the app and the illustrations were very nice to look at. They also appreciated the amount of text. One mother explained: *I think it was really easy to navigate in, it was very easy and like… for me, it was very easy to overview and it was nothing, because sometimes… things can be this very long and heavy but it was short, fairly short texts and so you could still move on and so, so I thought it was… no, it was easily accessible* (mother, no.9). However, two parents indicated wanted a clearer overview of the content, menus, and pages in the app because they sometimes got lost in it.

Five of the parents mentioned that the app gave a feeling of their grief being normal, that you are not alone in your grief, and you are not weird or crazy to feel the way that you feel because of the loss: *to feel this…well, I recognize that, then it's not just me who's weird* (mother, no. 11). It created a sense of belonging, and a confirmation that what you do and how you feel is totally okay, which generated a sense of normalization: *I think at least for me, this confirmation that it* [the app] *only exists or that… that an app like this is actually needed says that it's okay to feel the way you do in some way… I think that's the big thing* (mother, no. 24).

However, two parents, who lost their children 6 and 7 years ago, told during the interview that the app could provoke grief and memories again, which was difficult. But what was good was that you could control the use of the app yourself and could choose to shut it down. One parent told that because the app opened up some wounds, it takes time to use all of the app's different sections and its content, more than the 4 weeks during it was tested.

When asked if there was a need for this kind of self-help app for bereaved parents, all parents answered that the app is really needed. That it is good that the app is created and exists, and that the app gives a valuable overview that has been asked for from all the parents during their grieving process. Almost all of the interviewed parents (6/8) wished that the app had existed for them earlier in their grieving process: *I kind of think it's amazing that there will be such an app because there is little support, which sort of comes to you. It's just the Childhood Cancer Foundation, there was nothing in the hospital or connected to that… But it is important that information is provided, that this app exists, because I think it would be a huge help for many parents that lose a child* (mother, no. 9).

Some parents asked during the interview if they got to keep the app after the study was ended, and they were very happy when they knew that they could keep it.

### My Grief App-Survey

Nine participants completed the survey questions about the app. The perceived helpfulness of the app was rated moderate or greater by the majority of participants (see Table 3). The majority was moderately satisfied or very satisfied with the app overall, while one participant was only slightly satisfied with the app. Seven of the nine participants responded that the app met their expectations. Eight of nine participants did not have any technical problems, while for one person the app crashed once. All nine would recommend the app to parents in a similar situation.

**Table 3:** My Grief App-survey Scores.

**Bereaved parents, *n* = 9**	**Not at all**	**Slightly**	**Moderately**	**Very**	**Extremely**	**Mean (*SD*)**
Helping me learn about symptoms of complicated grief	–	3	4	1	1	2.0 (1.0)
Helping me finding effective ways of managing my grief	1	3	4	1	–	1.6 (0.9)
Helping me feel more comfortable in seeking help and support.	1	2	3	3	–	1.9 (1.1)
Helping me feel there is something I can do about my grief	1	1	3	4	–	2.1 (1.1)
Helping me track my grief over time	–	2	5	2	–	2.0 (0.7)
Helping me know when I'm doing better or when I'm doing worse	1	2	4	2	–	1.8 (1.0)
Providing practical solutions to the problems I experience.	1	4	2	2	–	1.6 (1.0)
Helping me better understand my grief reactions	1	1	5	2	–	1.9 (0.9)
Enhancing my knowledge of complicated grief.	–	2	3	3	1	2.3 (1.0)
Providing a way for me to talk about what I have been experiencing.	2	3	3	1	–	1.3 (1.0)

*Ratings. 0 = not at all; 1 = slightly; 2 = moderately; 3 = very; 4 = extremely. SD, Standard Deviation*.

Four participants responded ‘yes' on the question “Did you experience any stressful or negative consequences of using the app?” and on the follow-up question on what was most stressful they responded: (1) *It forces out emotions that I repress. Stressful but probably not negative*. (2) *Some formulations were provocative*. (3) *I could not use all the parts, it was too difficult emotionally*. (4) *Stressful not being able to do all the exercises, and not clear how to use the app day by day/step by step*.

### Prolonged Grief, Depression, PTSD and Cognitive Processes Related to the Loss

Among 10 participants filling out pre- and post-measures the Wilcoxon paired samples tests indicated that prolonged grief symptoms decreased from pre- to post-assessment (*Z* = −2.37, *p* = 0.018; *d* = 0.86), whereas symptoms of depression (*Z* = −1.15, *p* = 0.25; *d* = 0.44), and PTSD (*Z* = −0.97, *p* = 0.33; *d* = 0.26) did not (Table 2). Grief-related rumination (*Z* = −1.99, *p* = 0.047; *d* = 0.72), anxious avoidance (*Z* = −2.03, *p* = 0.042; *d* = 0.95) and negative grief cognitions (*Z* = −2.82, *p*= 0.005; *d* = 1.36) decreased from pre- to post-assessment, while there was no difference on depressive avoidance (*Z* = 1.08, *p*=.28; *d* = 0.37).

## Discussion

This pilot study showed that using the My Grief app was feasible and acceptable for bereaved parents. Participants reported satisfaction with the app and stated that they would recommend it to parents in similar situations. According to the participants, the app was easy to use, normalized grief reactions, and gave a valuable overview of information, knowledge, and further support. All four sections of the app were tested by the users, with predominantly positive experiences. The parents also had suggestions on how to improve the app. In addition, all parents expressed that an app like My Grief is needed and that it would be particularly useful to access early in the grieving process. Additionally, our open trial illustrated that access to the My Grief app may reduce prolonged grief symptoms and maladaptive cognitive-behavioral processes in bereaved parents. Notably, among 10 parents, the within-subject effect size was large for prolonged grief, which is comparable with previous studies examining the effect of treatment of PGD (10, 14). For example, Boelen and colleagues (10) reported large within-group effect sizes between pre- and post-treatment (*d* = 1.36–1.80) and Eisma and colleagues (14) reported moderate to large within-group effect sizes (*d* = 0.73–0.92).

When assessing the feasibility of a complex intervention in healthcare, one important part is the recipient's perspective on acceptability and delivery of the intervention (45, 46). Our study illustrated that the content in the app was considered useful, that the app was easy to use, and that it did not cause any harm for the participating parents. However, four parents reported some stressful or negative consequences of using the app such as provocative formulations and that it could be difficult emotionally to use it. It should be noted that confrontational elements, such as exposure, are an integral and effective part of CBT for PGD (10, 14). We therefore considered it necessary to offer exposure exercises to app users. Guidance by a therapist may help reduce the resistance to exposure exercises. An interesting option for future research is to evaluate the use of the My Grief app with therapist support, to help improve the implementation of exposure by participants. Some parents in this study came up with ideas on how to improve the app. Therefore, minor changes were made to the app, according to these suggestions, to improve the app before testing it in an RCT. For example, two parents expressed a feeling of “being lost” in the app, and they wanted a clearer overview of where one is in the app. Therefore, small adjustments were made to improve the navigation of the app, by adding a trail of links at the top of the page. Other users expressed a wish for reading more about other bereaved parents, how they coped with the situation and their grief, as well as tips on literature and podcast regarding grief. Therefore, the research group sent out an invitation to the parents that had tested the app after their participation in the study, and asked if they wanted to contribute to the content of the app with their own stories, which were added to the app. Additionally, the research group took the opportunity to adjust certain words in the texts describing grief, labeled as difficult to understand.

Some participants expressed that they did not use the app as much as they intended to, which is in line with other research on self-management smartphone apps for reducing mental illness (25, 47–49). Some of the parents in the current study expressed that the app would have been most helpful earlier in the grieving process. While the purpose of the app was to develop an app for parents with prolonged grief, the findings of this study suggest that parents with lower levels of prolonged grief also benefited from using the app. Therefore, it may be helpful to examine the usefulness of the app as a preventive intervention in future research.

Pilot testing also allows for establishing if methodological issues may arise in a subsequent a larger RCT, such as problems with recruitment, retention and data collection (50). We tested recruitment via the Swedish Childhood Cancer foundation's social media sites. However, during the recruitment process, a substantial minority of parents (8 of 27; 30%) were excluded because of responding “yes” to the question regarding suicidal thoughts. It should be considered that bereaved parents may have thoughts of death without necessarily wanting to commit suicide, which could result in people not receiving access to an app that may be helpful to them. The outcome assessment method is considered to be feasible and not too burdensome, as 13 of 14 participants who consented to participate completed the pre-assessment. Some parents told during the telephone interview that the pre-assessment had been helpful and validated their feelings regarding the loss and the feeling of not being alone in their grief experience. However, three participants dropped out (23%) and did not complete the post-assessment. The drop-out rate of below 25%, has been seen in other studies, such as those evaluating the PTSD Coach, and can be considered acceptable.

While this pilot study has generated promising results, the study has some limitations. First, the small sample size limits the power to adequately detect change over time in all types of symptoms of psychopathology and cognitive-behavioral processes. The non-significant results for small effects on depression and posttraumatic stress symptoms and depressive avoidance appear due to our limited sample size. A larger sample size may have yielded more consistent findings. Nevertheless, it could also be that the relatively low levels of symptoms of depression and posttraumatic stress and the short trial period of 4 weeks led to smaller effects, thereby reducing power. In addition, this pilot study lacked a control group so we do not know whether the changes in prolonged grief and cognitive-behavioral variables were due to access to the app or merely a result of natural recovery over time. However, considering that bereaved parents do not show much natural recovery over extended time-periods (51) we consider it likely that the effects can (at least partially) be attributed to the use of the app. Third, the app was used for a short period and cannot, therefore, completely reflect the intended real-world usage. Some parents expressed that it takes time to thoroughly evaluate the app's different parts, functions, and sections, as well as develop a routine in using the app, e.g., for self-monitoring of grief intensity. The lack of a control group and the short period of time for the users with the app (4 weeks) differs from the design of the larger planned RCT, which includes a wait-list control and app access for 3 months (28). The difference in design was because this pilot-study mainly focused on users' experiences and feasibility of the intervention. Fourth, a majority of the participating parents were highly educated women born in Sweden, so the sample is not representative of all parents who have lost a child. Lastly, we do not know whether the parents who dropped out and did not complete the post-assessment or took part in the interview, were less satisfied with the app than the completers, which may have affected the results.

Despite these limitations, this study offers unique insights. To our knowledge, this is the first study to examine the feasibility of a mobile app for prolonged grief. Additionally, it is the first feasibility study of an app supporting bereaved parents. Our pilot study indicates that the My Grief app is feasible and acceptable. Furthermore, participants showed a reduction in prolonged grief symptoms and most maladaptive cognitive-behavioral variables proposed to prolong grief. Based on the results from the current study, some minor modifications have been made to the app in line with suggestions by the participants and the recruitment process has been slightly modified for a planned RCT testing the effects of the My Grief app.

## Data Availability Statement

The raw data supporting the conclusions of this article will be made available by the authors, without undue reservation.

## Ethics Statement

The study has received ethical approval from the Swedish Ethical Review Authority (project no. 2020-01704). The participants provided their written informed consent to participate in this study. Written informed consent was obtained from the individual(s) for the publication of any potentially identifiable images or data included in this article.

## Author Contributions

RE, JS, ME, FA, and PB developed the study design. RE and JS performed the analyses and developed the draft of the manuscript. ME, FA, and PB read, revised, and approved the draft of the manuscript. All authors read and approved the final manuscript.

## Funding

This research was supported by the Swedish Childhood Cancer Foundation [grant numbers PR2018-0047, TJ2018-0002].

## Conflict of Interest

The authors declare that the research was conducted in the absence of any commercial or financial relationships that could be construed as a potential conflict of interest.

## Publisher's Note

All claims expressed in this article are solely those of the authors and do not necessarily represent those of their affiliated organizations, or those of the publisher, the editors and the reviewers. Any product that may be evaluated in this article, or claim that may be made by its manufacturer, is not guaranteed or endorsed by the publisher.

## Abbreviations

App: A smartphone application
CBT: Cognitive behavioral therapy
PGD: Prolonged grief disorder
PG13: Prolonged Grief Disorder-13
PTSD: Post-traumatic stress disorder
RCT: Randomized controlled trial.

## References

[1] Zhang B El-Jawahri A Prigerson HG. Update on bereavement research: evidence-based guidelines for the diagnosis and treatment of complicated bereavement. J Palliat Med. (2006) 5:1188–203. 10.1089/jpm.2006.9.1188 17040157

[2] October T Dryden-Palmer K Copnell B Meert KL. Caring for parents after the death of a child. Pediatr Crit Care. (2018) 8:61–8. 10.1097/PCC.0000000000001466 30080812 PMC6082144

[3] Pohlkamp L Kreicbergs U Sveen J. Bereaved mothers' and fathers' prolonged grief and psychological health 1-5 years after loss–a nationwide study. Psycho Oncol. (2019) 7:1530–36. 10.1002/pon.5112 31108000

[4] Kersting A Brähler E Glaesmer H Wagner B. Prevalence of complicated grief in a representative population-based sample. J Affect Disord. (2011) 131:339–43. 10.1016/j.jad.2010.11.032 21216470

[5] Morris S Fletcher K Goldstein R. The grief of parents after the death of a young child. J Clin Psychol Med Settings. (2019) 3:321–38. 10.1007/s10880-018-9590-7 30488260

[6] Boelen P Eisma MC Smid GE Lenferink L. Prolonged grief disorder in section II of DSM-5: a commentary. Eur J Psychotraumatol. (2020) 11:1. 10.1080/20008198.2020.1771008 33029316 PMC7473035

[7] Pohlkamp L Kreicbergs U Prigerson HG Sveen J. Psychometric properties of the Prolonged Grief Disorder-13 (PG-13) in bereaved Swedish parents. Psychiatry Res. (2018) 267:560–5. 10.1016/j.psychres.2018.06.004 29982112

[8] Kochen EM Jenken F Boelen PA Deben LMA Fahner JC van den Hoogen A. When a child dies: a systematic review of well-defined parent-focused bereavement interventions and their alignment with grief- and loss theories. BMC Palliat Care. (2020) 19:1. 10.1186/s12904-020-0529-z 32164668 PMC7068872

[9] Lövgren M Sveen J. Family bereavement care. In: Wolfe J, editor. Palliative Care in Pediatric Oncology. Cham: Springer (2018). p. 245–64.

[10] Boelen P de Keijser J van den Hout MA van den Bout J. Treatment of complicated grief: a comparison between cognitive-behavioral therapy and supportive counseling. J Consult Clin Psychol. (2007) 2:277–84. 10.1037/0022-006X.75.2.277 17469885

[11] Bryant RA Kenny L Joscelyne A Rawson N Maccallum F Cahill C. Treating prolonged grief disorder: a randomized clinical trial. JAMA Psychiatry. (2014) 12:1332–9. 10.1001/jamapsychiatry.2014.1600 25338187

[12] Rosner R Pfoh G Kotoučová M Hagl M. Efficacy of an outpatient treatment for prolonged grief disorder: a randomized controlled clinical trial. J Affect Disord. (2014) 167:56–63. 10.1016/j.jad.2014.05.035 25082115

[13] Shear K Frank E Houck PR Reynolds CF. Treatment of complicated grief: a randomized controlled trial. JAMA Psychiatry. (2005) 21:2601–8. 10.1001/jama.293.21.2601 15928281 PMC5953417

[14] Eisma M Boelen P van den Bout J Stroebe W Schut HA Lancee J. Internet-based exposure and behavioral activation for complicated grief and rumination: a randomized controlled trial. Behav Ther. (2015) 6:729–48. 10.1016/j.beth.2015.05.007 26520217

[15] Kersting A Dölemeyer R Steinig J Walter F Kroker K Baust K. Brief internet-based intervention reduces posttraumatic stress and prolonged grief in parents after the loss of a child during pregnancy: a randomized controlled trial. Psychother Psychosom. (2013) 6:372–81. 10.1159/000348713 24061387

[16] Treml J Nagl M Linde K Kündiger C Peterhänsel C Kersting A. Efficacy of an Internet-based cognitive-behavioural grief therapy for people bereaved by suicide: a randomized controlled trial. Eur J Psychotraumatol. (2021) 12:1926650. 10.1080/20008198.2021.1926650 34992754 PMC8725716

[17] Doering BK. Eisma MC. Treatment for complicated grief: state of the science and ways forward. Curr Opin Psychiatry. (2016) 5:286–91. 10.1097/YCO.0000000000000263 27429216

[18] Knowles LM Jovel KS Mayer CM Bottrill KC Kaszniak AW Sbarra DA. A controlled trial of two mind–body interventions for grief in widows and widowers. J Consult Clin Psychol. (2021) 7:640–54. 10.1037/ccp0000653 34383536

[19] Lenferink L de Keijser J Wessel I Boelen PA. Cognitive behavioural therapy and mindfulness for relatives of missing persons: a pilot study. Pilot Feasibi Stud. (2019) 1:1–17. 10.1186/s40814-019-0472-z 31363418 PMC6642737

[20] O'Connor M Piet J Hougaard E. The effects of mindfulness-based cognitive therapy on depressive symptoms in elderly bereaved people with loss-related distress: a controlled pilot study. Mindfulness. (2014) 5:400–409. 10.1007/s12671-013-0194-x

[21] Olff M. Mobile mental health: a challenging research agenda. Eur J Psychotraumatol. (2015) 6:27882. 10.3402/ejpt.v6.27882 25994025 PMC4439418

[22] Wang K Varma SD Prosperi M. A systematic review of the effectiveness of mobile apps for monitoring and management of mental health symptoms and disorders. J Psychiatr Res. (2018) 107:73–8. 10.1016/j.jpsychires.2018.10.006 30347316

[23] Kuhn E van der Meer C Owen JE Hoffman JE Cash R Carrese P. PTSD Coach around the world. Mhealth. (2018) 4:15. 10.21037/mhealth.2018.05.01 29963560 PMC5994444

[24] Hensler I Sveen J Cernvall M Arnberg FK. Access to PTSD coach decreases posttraumatic stress and depressive symptoms: randomized controlled trial of efficacy, benefits and harms of a self-management app in a Swedish, trauma-exposed community sample. J Med Internet Res. (2022) 22. 10.2196/preprints.31419 PMC9008528 35353052

[25] Cernvall M Sveen J Bergh Johannesson K Arnberg F. A pilot study of user satisfaction and perceived helpfulness of the Swedish version of the mobile app PTSD Coach. Eur J Psychotraumatol. (2018) 9:1472990. 10.1080/20008198.2018.1472990 29805783 PMC5965023

[26] Kuhn E Greene C Hoffman J Nguyen T Wald L Schmidt J. Preliminary evaluation of PTSD Coach, a smartphone app for post-traumatic stress symptoms. Mil Med. (2014) 1:12–18. 10.7205/MILMED-D-13-00271 24402979

[27] Miner A Kuhn E Hoffman JE Owen JE Ruzek JI Taylor CB. Feasibility, acceptability, and potential efficacy of the PTSD Coach app: a pilot randomized controlled trial with community trauma survivors. Psychol Trauma Theory Res Pract Policy. (2016) 3:384–92. 10.1037/tra0000092 27046668

[28] Eklund R Eisma M Boelen P Arnberg F Sveen J. Mobile app for prolonged grief among bereaved parents: study protocol for a randomized controlled trial. BMJ Open. (2021) 11. 10.1101/2021.04.23.21256003 34876429 PMC8655571

[29] Boelen P Van Den Hout MA Van Den Bout J. A cognitive-behavioral conceptualization of complicated grief. Clin Psychol Sci Pract. (2006) 2:109–28. 10.1111/j.1468-2850.2006.00013.x15493080

[30] Lenferink L de Keijser J Eisma M Smid G Boelen P. Online cognitive–behavioural therapy for traumatically bereaved people: study protocol for a randomised waitlist-controlled trial. BMJ Open. (2020) 10:e035050. 10.1136/bmjopen-2019-035050 32883723 PMC7473627

[31] Reitsma L Boelen PA de Keijser J Lenferink LIM. Online treatment of persistent complex bereavement disorder, posttraumatic stress disorder, and depression symptoms in people who lost loved ones during the COVID-19 pandemic: study protocol for a randomized controlled trial and a controlled trial. Eur J Psychotraumatol. (2021) 12:1987687. 10.1080/20008198.2021.1987687 34868479 PMC8635653

[32] Prigerson HG Horowitz MJ Jacobs SC Parkes CM Aslan M Goodkin K. Prolonged grief disorder: psychometric validation of criteria proposed for DSM-V and ICD-11. PLoS Med. (2009) 6:8. 10.1371/journal.pmed.1000121 19652695 PMC2711304

[33] Gilbody S Richards D Brealey S Hewitt C. Screening for depression in medical settings with the Patient Health Questionnaire (PHQ): a diagnostic meta-analysis. J Gen Intern Med. (2007) 22:11:1596–602. 10.1007/s11606-007-0333-y 17874169 PMC2219806

[34] Kroenke K Spitzer RL Williams JB. The PHQ-9: validity of a brief depression severity measure. J Gen Intern Med. (2001) 9:606–13. 10.1046/j.1525-1497.2001.016009606.x 11556941 PMC1495268

[35] Blevins CA Weathers FW Davis MT Witte TK Domino JL. The posttraumatic stress disorder checklist for DSM-5 (PCL-5): development and initial psychometric evaluation. J Trauma Stress. (2015) 6:489–98. 10.1002/jts.22059 26606250

[36] The National Centre for PTSD. The PTSD Checklist for DSM-5 (PCL-5). (2013). https://www.ptsd.va.gov (accessed January 28, 2022).

[37] Sveen J Bondjers K Willebrand M. Psychometric properties of the PTSD Checklist for DSM-5: a pilot study. Eur J Psychotraumatol. (2016) 7:30165. 10.3402/ejpt.v7.30165 27098450 PMC4838990

[38] Eisma MC Stroebe MS Schut HA Van Den Bout J Boelen PA Stroebe W. Development and psychometric evaluation of the Utrecht Grief Rumination Scale. J Psychopathol Behav Assess. (2014) 1:165–76. 10.1007/s10862-013-9377-y

[39] Sveen J Pohlkamp L Kreicbergs U Eisma MC. Rumination in bereaved parents: psychometric evaluation of the Swedish version of the Utrecht Grief Rumination Scale (UGRS). PLoS ONE. (2019) 3:e0213152. 10.1371/journal.pone.0213152 30889209 PMC6424480

[40] Boelen P Van den Bout J. Anxious and depressive avoidance and symptoms of prolonged grief, depression, and post-traumatic stress disorder. Psychol Belg. (2010) 1–2:49–67. 10.5334/pb-50-1-2-49

[41] Kulis D Bottomley A Velikova G Greimel E Koller M EORTC. Quality of Life Group Translation Procedure. European Organization for Research and Treatment of Cancer (2017).

[42] Doering BK Boelen PA Eisma MC Barke A. Validation of a German version of the grief cognitions questionnaire and establishment of a short form. Front Psychol. (2020) 11:620987. 10.3389/fpsyg.2020.620987 33536985 PMC7848142

[43] Braun V Clarke V. Using thematic analysis in psychology. Qual Res Psychol. (2006) 2:77–101. 10.1191/1478088706qp063oa

[44] Morris SB DeShon RP. Combining effect size estimates in meta-analysis with repeated measures and independent-groups designs. Psychol Methods. (2002) 1:105–25. 10.1037/1082-989X.7.1.105 11928886

[45] Campbell M Fitzpatrick R Haines A Kinmonth AL Sandercock P Spiegelhalter D. Framework for design and evaluation of complex interventions to improve health. Bmj. (2000) 321:694–6. 10.1136/bmj.321.7262.694 10987780 PMC1118564

[46] Craig P Dieppe P Macintyre S Michie S Nazareth I Petticrew M. Developing and evaluating complex interventions: the new Medical Research Council guidance. BMJ. (2008) 337:a1655. 10.1136/bmj.a1655 18824488 PMC2769032

[47] Owen JE Jaworski BK Kuhn E Makin-Byrd KN Ramsey KM Hoffman JE. mHealth in the wild: using novel data to examine the reach, use, and impact of PTSD coach. JMIR Ment Health. (2015) 2:e7. 10.2196/mental.3935 26543913 PMC4607374

[48] Peng W Kanthawala S Yuan S Hussain SA. A qualitative study of user perceptions of mobile health apps. BMC Public Health. (2016) 16:1158. 10.1186/s12889-016-3808-0 27842533 PMC5109835

[49] Riisager LH Christensen AB Scharff FB Arendt I-MT Ismail I Lau ME. Patients' experiences of using a self-help app for posttraumatic stress disorder: qualitative study. JMIR Form Res. (2021) 8:e26852. 10.2196/26852 34346896 PMC8374664

[50] Taylor RS Ukoumunne OC Warren FC. How to use feasibility and pilot trials to test alternative methodologies and methodological procedures prior to full-scale trials. In: Richards DA, Rahm Hallberg I, editors. Complex Interventions in Health, An Overview of Research Methods. New York, NY: Routledge (2015). 136–44.

[51] Buyukcan-Tetik A Albuquerque S Stroebe MS Schut HA Eisma MC. Grieving together: dyadic trajectories and reciprocal relations in parental grief after child loss. In: Scott CL, Williams HM, Wilder S, editors. Contemporary Perspectives in Family Research Series, Facing Death: Familial Responses to Illness and Death. Bingley: Emerald Publishing (2022).

