# Supplementary material for: My Grief App for Prolonged Grief in Bereaved Parents: A Pilot Study
Source: Front Psychiatry. 2022 Apr 25;13:872314. doi: 10.3389/fpsyt.2022.872314 (PMC9082261; doi:10.3389/fpsyt.2022.872314)
Supplement: Supplementary file 1 [file Data_Sheet_1.DOCX]

**Interview guide for evaluation of the My Grief app**

**Overall questions**

Can you please start by telling me a little about how you came in contact with the research project and the app?

Please, tell me briefly how you used the app overall.

The app consists of four sections “learn”, “self-monitoring of grief intensity”, “exercises”, and “get support”.

Have you looked through all the sections?

If not: which? Why?

Which section did you like the most? Why?

**Questions regarding section 1: “learn”**

Did you learn something new regarding grief?

If yes: what?

Which part in the section “learn” was most beneficial for you? Why?

**Questions regarding section 2: “self-monitoring of grief intensity”**

Did you register your grief daily?

If not: why?

In what way was this section helpful?

**Questions regarding section 3: “exercises”**

There are a bunch of different exercises in the app, both those you can listen to, read yourself or write.

Which exercise did you use the most? Why?

Which exercise did you like the most? Why?

Was there any exercise that was difficult to perform?

If yes: Why?

Was there any exercise that was emotionally hard to perform?

If yes: Why?

How much time per week did you spend doing these exercises?

How many different exercises did you do?

**Questions regarding section 4: “get support”**

What did you think about this section?

Did you get help from any link or helpline?

If yes: Which? Why?

Did you learn anything from the information texts in the section?

If yes: what?

**Some general questions about the app**

Approximately how many times during a week did you use the app?

How did you experience the app’s layout?

Colors, icons, amount of text

Did you have any technical problems with the app?

With the installations, bugs

Was there something you were missing in the app?

Is there anything we can do to improve the app?

What was the most positive thing about the app?

Is there something I forgot to ask about the app that you would like to tell me?
